# Precuneus and Cingulate Cortex Atrophy and Hypometabolism in Patients with Alzheimer's Disease and Mild Cognitive Impairment: MRI and ^18^F-FDG PET Quantitative Analysis Using FreeSurfer

**DOI:** 10.1155/2015/583931

**Published:** 2015-06-17

**Authors:** Matthieu Bailly, Christophe Destrieux, Caroline Hommet, Karl Mondon, Jean-Philippe Cottier, Emilie Beaufils, Emilie Vierron, Johnny Vercouillie, Méziane Ibazizene, Thierry Voisin, Pierre Payoux, Louisa Barré, Vincent Camus, Denis Guilloteau, Maria-Joao Ribeiro

**Affiliations:** ^1^Service de Médecine Nucléaire, CHRU de Tours, boulevard Tonnellé, 37000 Tours, France; ^2^Service de Médecine Nucléaire, CHR Orléans, avenue de l'Hôpital, 45000 Orléans, France; ^3^INSERM U930, Université François Rabelais, boulevard Tonnellé, 37000 Tours, France; ^4^Consultation Mémoire, CHRU de Tours, boulevard Tonnellé, 37000 Tours, France; ^5^Service de Neuroradiologie, CHRU de Tours, boulevard Tonnellé, 37000 Tours, France; ^6^INSERM CIC 1415, boulevard Tonnellé, 37000 Tours, France; ^7^CEA/DSV/I2BM/CI-NAPS UMR6232, 14000 Caen, France; ^8^INSERM U825, Université Paul Sabatier, 31400 Toulouse, France

## Abstract

*Objective.* The objective of this study was to compare glucose metabolism and atrophy, in the precuneus and cingulate cortex, in patients with Alzheimer's disease (AD) and mild cognitive impairment (MCI), using FreeSurfer. *Methods.* 47 individuals (17 patients with AD, 17 patients with amnestic MCI, and 13 healthy controls (HC)) were included. MRI and PET images using ^18^F-FDG (mean injected dose of 185 MBq) were acquired and analyzed using FreeSurfer to define regions of interest in the hippocampus, amygdala, precuneus, and anterior and posterior cingulate cortex. Regional volumes were generated. PET images were registered to the T1-weighted MRI images and regional uptake normalized by cerebellum uptake (SUVr) was measured. *Results.* Mean posterior cingulate volume was reduced in MCI and AD. SUVr were different between the three groups: mean precuneus SUVr was 1.02 for AD, 1.09 for MCI, and 1.26 for controls (*p* < 0.05); mean posterior cingulate SUVr was 0.96, 1.06, and 1.22 for AD, MCI, and controls, respectively (*p* < 0.05). *Conclusion.* We found graduated hypometabolism in the posterior cingulate cortex and the precuneus in prodromal AD (MCI) and AD, whereas atrophy was not significant. This suggests that the use of ^18^F-FDG in these two regions could be a neurodegenerative biomarker.

## 1. Introduction

New diagnostic criteria have been introduced in Alzheimer's disease (AD) (Dubois et al. [1] and McKhann et al. [2] and more recently the IWG-2 criteria [3]). They suggest that the diagnosis of “prodromal AD” (also called the AD predementia stage) [4] or “mild cognitive impairment (MCI) due to AD pathology” [2] should rely on in vivo biomarkers. Biomarkers of amyloid pathology (amyloid PET tracers, cerebrospinal fluid (CSF) dosage of A*β*) and biomarkers of degenerative neurofibrillary tangles (CSF dosage of tau and phosphorylated tau, temporoparietal hypometabolism on ^18^F-fluorodeoxyglucose (FDG) PET, and medial temporal atrophy on MRI) can now provide an earlier diagnosis, especially in young subjects and atypical profiles.

Cerebral atrophy typically starts in the medial temporal and limbic areas, spreads to parietal association areas, and finally progresses to frontal and primary cortices [5]. For many years the medial temporal lobe, especially the hippocampus, was the focal point for the early diagnosis of AD, for example, using Scheltens's evaluation [6]. ^18^F-FDG also assesses neurodegeneration by measuring cerebral metabolic rate of glucose (CMRglc). AD is characterized by a particular regional pattern of low CMRglc: the posterior cingulate cortex is the first area affected and is followed by the parietotemporal areas (especially the hippocampus) and then the frontal regions as the disease progresses [7].

It is important to notice that diagnostic and prognostic accuracy of imaging biomarkers is at least as dependent on how the biomarker is measured as it is on the type of biomarker itself. There is a wide range of metrics, from visual analysis to quantitative analysis based on manual segmentation or automatic segmentation. Visual reading or manual regions of interest (ROIs) tracing induces interobserver variability. FreeSurfer is a free automated structural MRI image processing software providing many anatomical measures, including gray matter volume (http://surfer.nmr.mgh.harvard.edu/) [8], and already used to measure atrophy in patients with AD or mild cognitive impairment (MCI) [9, 10]. It has also been used to analyze and quantify PET data [11, 12].

PET and MRI biomarkers of neuronal injury focus on the medial temporal region. Both precuneus and cingulate cortex show prominent reduced metabolism on ^18^F-FDG PET and atrophy on MRI and could also be considered as one of the biomarkers [13]. The present study aims to analyze regional CMRglc (as assessed by ^18^F-FDG PET) with atrophy (as assessed by MRI) in precuneus and cingulate cortex of patients with AD or prodromal AD (MCI) and healthy controls (HC) from the elderly population, using the same automated software, FreeSurfer.

## 2. Materials and Methods

### 2.1. Patients and Controls

Forty-seven individuals (seventeen patients with AD, seventeen patients with amnestic MCI, and thirteen healthy controls (HC)) were recruited in this study. All patients were referred to one of the three participating memory clinics of the University Hospitals of Tours, Caen, and Toulouse in France. They were at least 55 years old, spoke fluent French, had completed at least seven years of education, and had neither unstable somatic disease nor psychiatric comorbidities (Table 1). Both MCI and AD patients were significantly older than HC.

Patients with AD were included according to the NINCDS-ADRDA criteria for probable AD [14] and the DSM-IV criteria for Alzheimer's type dementia. New diagnostic criteria [2] were not published at the time of their recruitment. Patients were required to have a Mini-Mental State Examination (MMSE) score between 15 and 28 [15]. They were not included if they had received (or were currently receiving) any symptomatic treatment with acetylcholinesterase inhibitors or memantine or if they had participated in any experimental study involving A*β*-lowering agents.

MCI patients met the diagnostic criteria for amnestic MCI [16]: they had a subjective memory complaint associated with isolated impairment in episodic memory and had a total recall below 40 during the free and cued recall test.

HC did not have any of the following: history of (or any current) major depressive episodes and/or antidepressant treatment; cognitive impairment as assessed by a neuropsychological battery (which evaluates episodic memory, language, praxia, gnosia, and executive functions); memory complaints; or MRI brain scan abnormalities.

The regional ethic committee of Tours (Comité de Protection des Personnes de la Région Centre) and the French Agency for Safety and Security of Medical Devices (AFSSAPS) approved the study protocol. Written informed consent was obtained from all persons participating in the study.

### 2.2. Brain Imaging

All participants underwent a brain MRI scan using a 1.5 T imager at one center (Tours) and 3 T imagers at the other two centers (Caen and Toulouse). T2-weighted images from each subject were used to investigate brain lesions. 3D T1-weighted volume (SPGR sequence, TE/TR/TI = 3.5/10.76/600 ms, bandwidth = 97 Hz/px, flip angle = 10°, and voxel size: 1.1 × 1.1 × 1.2 mm^3^) was used for volumetric analysis.

PET-CT scans with ^18^F-FDG were performed on a hybrid tomograph, operating in 3D detection mode (Dual Gemini, Philips Medical Systems, Discovery RX VCT 64, General Electric, and Biograph 6 TruePoint HiRez, Siemens Medical Solutions, resp., in Tours, Caen, and Toulouse). Capillary glycaemia was checked prior to ^18^F-FDG PET. PET scans were acquired 30 minutes after an injection of 185 MBq of ^18^F-FDG (mean value). A dynamic PET acquisition was performed, lasting 30 minutes with six 5 min frames. All PET sinograms were reconstructed by adapting the parameters to those of the tomograph with the lowest spatial resolution (Dual Gemini), with corrections for randomness, scatter, photon attenuation, and decay, which produced images with an isotropic voxel of 2 × 2 × 2 mm^3^. The six frames were averaged into a single image.

### 2.3. Image Analysis

Volumetric segmentation was performed with the FreeSurfer image analysis suite (http://surfer.nmr.mgh.harvard.edu/) [8]. Briefly, this processing includes the following: removal of nonbrain tissue using a hybrid watershed/surface deformation procedure; automated Talairach transformation; segmentation of the subcortical white matter and deep gray matter volumetric structures; intensity normalization; tessellation of the gray matter white matter boundary; automated topology correction; and surface deformation using intensity gradients to optimally place the gray/white and gray/cerebrospinal fluid borders at the position of the greatest shift in intensity, which defines the transition between tissue classes.

This software generated many cortical volumes, surface areas, and cortical thicknesses, as well as other values. We focused on the following ROIs: total brain, hippocampus, amygdala, precuneus, anterior cingulate cortex, posterior cingulate cortex, and cerebellum. This segmentation approach has been used for multivariate classification of AD and HC [17], MCI volumetric studies [9, 10], neuropsychological-image analysis [18, 19], imaging-genetic analysis [20, 21], and biomarker discovery [22]. For each region (hippocampus, amygdala, precuneus, anterior cingulate cortex, and posterior cingulate cortex), we summed the right and the left volume, and we normalized these volumes by the total intracranial volume without ventricles, similar to a recent study [23].

**Figure 1:**
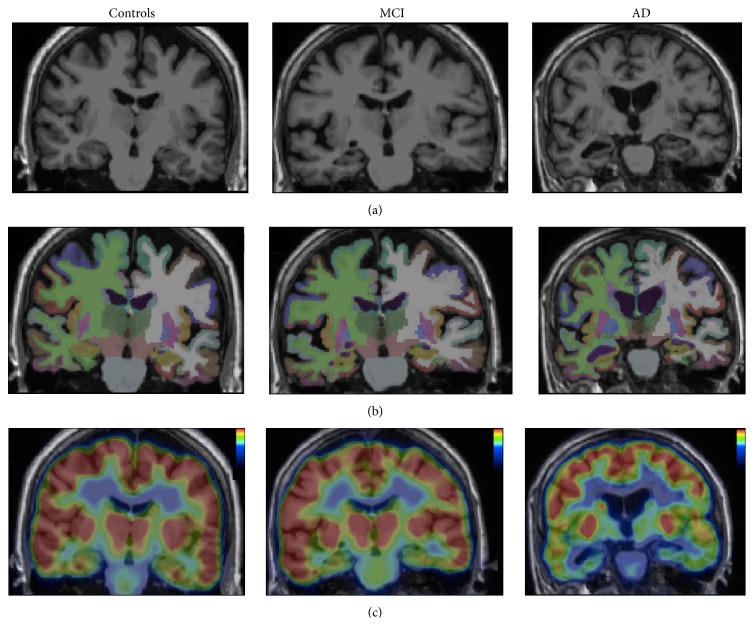
Coronal view of MRI T1-weighted image (a), segmentation of the brain in FreeSurfer (b), and ^18^F-FDG PET registered on MRI (c) for each group.

After correction of motion, ^18^F-FDG averaged images were automatically aligned to the MRI T1-weighted scans, using the Boundary-Based Registration algorithm in FreeSurfer, which involves a linear transformation with six degrees of freedom. The registration accuracy was assessed visually for each subject and the FreeSurfer segmentations were mapped into the PET space (Figure 1). We directly measured maximal PET intensities in the ROIs already defined. The regional to cerebellum ratios (SUVr) were used for interindividual comparisons because the cerebellum has been reported to be a region in which CMRglc is least affected in AD [24, 25].

### 2.4. Statistical Analysis

Statistical analysis was performed using R software [26]. Descriptive statistics and tests were separately computed for the AD, MCI, and control subjects. The chi-squared test was applied to assess differences in gender between groups. Age, MMSE, ^18^F-FDG, and MRI data were analyzed separately using a global Kruskal-Wallis nonparametric test. When significant global *p* values were obtained, pairwise comparisons between the three groups were performed using the Wilcoxon-Mann-Whitney test, and *p* values were adjusted using the Benjamini and Hochberg method [27]. Spearman's correlations were used to assess bivariate relationships between MRI atrophy and ^18^F-FDG hypometabolism. All tests were two-sided. Results were considered significant for *p* < 0.05.

## 3. Results

### 3.1. Volume Analysis

The mean hippocampal volume normalized by total brain volume without ventricles (± standard deviation (SD)), as determined by FreeSurfer, was different between the three groups: 0.574% (±0.095) for patients with AD, 0.636% (±0.099) for patients with MCI, and 0.747% (±0.062) for HC (*p* < 0.001). Mean hippocampal volume was significantly smaller in the AD group and in the MCI group than in the control elderly population (*p* < 0.001 and *p* < 0.05) (Table 2).

We also observed a difference between the three groups regarding the posterior cingulate volume (*p* < 0.05). Finally, mean amygdala volume was smaller both in the AD and in the MCI group than in the HC group, but there was no significant difference.

### 3.2. Assessment of Cerebral Metabolic Rate of Glucose

We found differences in hippocampal SUVr between the three groups as assessed by FreeSurfer, with a significant lower hippocampal SUVr in AD subjects than controls (*p* < 0.01): the mean hippocampus to cerebellum ratio (± SD) was 0.69 ± 0.10 for patients with AD, 0.75 ± 0.13 for patients with MCI, and 0.84 ± 0.10 for HC. There were no significant differences between patients with AD and patients with MCI or between patients with MCI and HC for the hippocampus. We also observed a trend towards low SUVr in the amygdala of patients with MCI or AD, although no significant difference was found (*p* = 0.05) (Table 3).

Besides this, we observed significant differences in CMRglc between the three groups: mean precuneus SUVr was 1.02 for patients with AD, 1.09 for MCI patients, and 1.26 for controls (*p* < 0.05). Mean posterior cingulate SUVr was 0.96, 1.06, and 1.22 for patients with AD, MCI patients, and controls, respectively (*p* < 0.05). Significantly smaller ^18^F-FDG uptake was found in the precuneus (*p* < 0.001) and in the anterior and posterior cingulate cortex (*p* < 0.001) both in MCI and in AD patients than in HC. Only precuneus and posterior cingulate regions showed reduced CMRglc in AD patients, compared to the MCI group (*p* < 0.05).

### 3.3. Comparison of Volume and CMRglc Regional Patterns

We conducted correlation analysis between regional ^18^F-FDG SUVr and MRI relative volume and observed a significant correlation between the CMRglc and atrophy in the hippocampus of AD patients (rho: 0.516, *p* < 0.05). No other significant correlation was found.

## 4. Discussion

The main objective of this study was to compare regional hypometabolism with atrophy in precuneus and cingulate cortex of patients with AD or prodromal AD, using the same automated software, FreeSurfer. Several morphological studies have analyzed MRI data using this software for the purpose of classifying AD or to predict the conversion of MCI to AD [9, 10, 23, 28]. Few recent studies used FreeSurfer ROIs to quantify the buildup of amyloid [11, 29]. However, few studies focused on the use of FreeSurfer to analyze ^18^F-FDG data [30].

In our study, hippocampal and posterior cingulate volumes were lower in the MCI and AD groups than in HC. Regarding CMRglc, there was significant hypometabolism in the precuneus and posterior cingulate cortex of AD patients compared to MCI subjects and HC and also in MCI patients compared to HC. Anterior cingulate cortex only showed a lower ^18^F-FDG uptake in AD and MCI patients than in HC, but there was no significant difference between AD and MCI subjects. We also observed a lower CMRglc in the hippocampus of AD patients than in HC. These data are consistent with the actual knowledge of atrophy and low CMRglc in the temporal regions of patients with AD, especially the hippocampus [31, 32]. It is well established that mesial temporal regions are relevant locations to look for metabolic or morphometric biomarkers for the early detection of AD, because neurofibrillary tangles first occur in these regions before spreading to other cortical areas [5, 33]. We also confirmed atrophy in the posterior cingulate cortex in AD subjects, and we showed reduction of CMRglc in the posterior cingulate cortex and in the precuneus in AD patients compared to MCI subjects and HC, consistent with previous findings [23, 33–35]. Karow et al. also suggested that ^18^F-FDG measures were redundant with MR imaging; according to them, a loss of tissue rather than a reduction in metabolism per unit of remaining tissue volume accounted for many of the effects observed with ^18^F-FDG PET [33]. Our findings showed that precuneus and posterior cingulate CMRglc are much more correlated to the disease status (MCI or AD) than atrophy assessed by MRI in our patients, suggesting that CMRglc in these two regions (precuneus and posterior cingulate cortex) could be considered as a neurodegenerative biomarker.

To investigate CMRglc, especially in the entorhinal cortex and the hippocampus, careful registration of ^18^F-FDG PET to MR images should be applied, combined with ROI-based analysis or voxel-based morphometric methods [32]. Manual ROI tracing induces interobserver variability, and automated structural processing should be preferred. Many studies report that FreeSurfer is a reliable method to determine segmentation from MRI in patients with AD or MCI [9–11] and is substantially more reliable than manual segmentation. Variability does however exist in the automated segmentation procedure [36–38], and manual correction of FreeSurfer derived boundaries is sometimes necessary, especially in marked atrophic brains. All our analyses were performed on the same computer and visually assessed by one physician, using the same version of the software, as Gronenschild et al. showed that variability could exist between different FreeSurfer versions or workstation types [39].

Nonetheless, Su et al. used FreeSurfer to quantify C11-PiB and found that this method was highly reliable for the estimation of regional measurements, despite variability in ROI volumes [11]. A recent study concluded that FreeSurfer could be used for quantification of PET data, using amyloid-beta and receptor 5-HT_1B_ radioligands [12]. This suggests the FreeSurfer-based PET quantification is reliable in many regions. One of the advantages of FreeSurfer is that it incorporates a comprehensive anatomical analysis, which can be applied to PET data. For instance, subregional PET analysis is automatically available with FreeSurfer and can be quickly obtained.

We chose to normalize regional ^18^F-FDG uptake by the cerebellar uptake, because the cerebellum has been reported to be a region in which CMRglc is least affected in AD [24, 25]. Other regions have been described as reliable references for data normalization: pons [40] or cerebral global normalization which might be superior for differential diagnostic purposes in dementia syndromes [41]. Küntzelmann et al. examined alterations of cerebral glucose metabolism in AD and prodromal AD depending on intensity normalization. They proved that cerebellar normalization was superior in differentiating patients with AD or prodromal AD from healthy controls. Global normalization provided slightly better contrasts for the differentiation between AD and prodromal AD in AD-typical regions in their study, but unexpected hypermetabolism in patients was only revealed using global normalization [42].

Regarding MRI volumes, we chose to normalize regional volumes by the total brain volume without the ventricles, to account for interindividual variations. Similar to Walhovd et al., we found that this normalization was the most commonly used one in the literature [43]. Several studies have analyzed different methods of MRI normalization. Westman et al. concluded that cerebral volumes should be normalized to intracranial volume [23]. Normalizing cortical volumes facilitated the discrimination between patients with AD, those with amnestic MCI, and controls in their study. However, this method has some weaknesses: the nonlinear relationship between brain volumes and intracranial volume [44] and the fact that the maximal brain size seems to be an important predictor of cognition in old age, independent of brain pathology [45]. Nonetheless we agree with Westman et al. that changes in neurodegenerative disorders are relatively small and may be overlooked if the data are not normalized [23].

Higher field strengths are becoming more commonly used clinically and in therapeutic trials. Chow et al. suggested that 3 T MRI images may be able to detect volume differences that are not apparent at 1.5 T [46]. MRI data were acquired at 1.5 T in one of the three centers; this could have introduced bias in favor of PET.

Our study has some limitations. Firstly, we included a small number of patients and controls; secondly, there was no histopathological verification of disease status; thirdly we did not apply partial volume correction, but we registered PET data to MRI and used intensities ratio to cerebellum; finally, MCI and AD patients were younger than controls. Nonetheless, FreeSurfer has several advantages: the analysis is a fully automated, accurate, and simple process and offers the option of only analyzing grey matter, thus excluding white matter and its nonspecific uptake (shown, e.g., by the uptake of florbetapir in amyloid density studies) [47].

## 5. Conclusion

In summary, the present study, using the same automated software, FreeSurfer, to analyze both ^18^F-FDG and MRI images, confirmed hippocampal atrophy in both AD and MCI patients and hypometabolism in the hippocampus, the cingulate cortex, and the precuneus of AD and MCI subjects. Moreover, our results showed a gradation in the decrease of CMRglc in the posterior cingulate cortex and the precuneus in prodromal AD (or MCI) and AD, suggesting the use of ^18^F-FDG in these two regions as a neurodegenerative biomarker, which could be positive earlier than MRI atrophy in those areas.

## Figures and Tables

**Table 1:** Demographic data and cognitive ability evaluated by MMSE for all groups.

	AD *n* = 17	HC *n* = 13	MCI *n* = 17
Sex (female)			
*n* (%)	10 (58.8)	8 (61.5)	9 (52.9)
Age (years)			
Mean (SD)	70.6 (5.7)	64.1 (3.3)	73.8 (8.1)
Median (min–max)	72.0 [60.0–81.0]	64.0 [60.0–68.0]	73.0 [59.0–89.0]
MMSE score			
Mean (SD)	21.7 (3.3)	29.2 (0.7)	26.1 (2.4)
Median (min–max)	21.5 [16.0–28.0]	29.0 [28.0–30.0]	27.0 [22.0–30.0]

AD: Alzheimer's disease; MCI: mild cognitive impairment; MMSE: Mini-Mental State Examination.

**Table 2:** MRI relative volume (%) for each region and group.

	AD *n* = 17	HC *n* = 13	MCI *n* = 17	*p*			
				Global	AD versus HC	AD versus MCI	MCI versus HC
Hippocampus							
Mean (SD)	0.574 (0.095)	0.747 (0.062)	0.636 (0.099)	0.00005	0.00003	0.05293	0.0013
Median (min–max)	0.591 [0.34–0.732]	0.764 [0.601–0.82]	0.648 [0401–0.819]				
Amygdala							
Mean (SD)	0.234 (0.044)	0.276 (0.043)	0.251 (0.058)	0.06152
Median (min–max)	0.234 [0.148–0.337]	0.271 [0.21–0.362]	0.245 [0.145–0.366]	
Precuneus							
Mean (SD)	1.557 (0.116)	1.532 (0.105)	1.47 (0.246)	0.85693			
Median (min–max)	1.511 [1.366–1.77]	1.522 [1.377–1.675]	1.539 [0.816–1.755]				
Caudal anterior cingulate							
Mean (SD)	0.323 (0.054)	0.333 (0.054)	0.319 (0.072)	0.86231			
Median (min–max)	0.322 [0.207–0.442]	0.341 [0.252–0.416]	0.316 [0.165–0.447]				
Rostral anterior cingulate							
Mean (SD)	0.415 (0.06)	0.378 (0.068)	0.379 (0.089)	0.37687			
Median (min–max)	0.414 [0.296–0.511]	0.386 [0.248–0.477]	0.406 [0.126–0.481]				
Isthmus cingulate							
Mean (SD)	0.43 (0.035)	0.401 (0.048)	0.398 (0.065)	0.17212			
Median (min–max)	0.425 [0.364–0.488]	0.399 [0.331–0.503]	0.401 [0.256–0.488]				
Posterior cingulate							
Mean (SD)	0.509 (0.042)	0.543 (0.025)	0.503 (0.095)	0.04422	0.05841	0.97289	0.05841
Median (min–max)	0.513 [0.443–0.579]	0.542 [0.504–0.589]	0.509 [0.268–0.68]				

AD: Alzheimer's disease; MCI: mild cognitive impairment.

Regional volumes are means of both right and left structures.

**Table 3:** ^18^F-FDG cerebral metabolic rate of glucose (SUVr) for each region and group.

	AD *n* = 17	HC *n* = 13	MCI *n* = 17	*p*			
				Global	AD versus HC	AD versus MCI	MCI versus HC
Hippocampus							
Mean (SD)	0.69 (0.10)	0.84 (0.10)	0.75 (0.13)	0.00227	0.00119	0.19004	0.06322
Median (min–max)	0.71 [0.40–0.83]	0.82 [0.71–1.11]	0.75 [0.45–1.02]				
Amygdala							
Mean (SD)	0.64 (0.11)	0.77 (0.17)	0.71 (0.19)	0.05145
Median (min–max)	0.67 [0.35–0.77]	0.71 [0.66–1.31]	0.69 [0.39–1.33]	
Precuneus							
Mean (SD)	1.02 (0.09)	1.26 (0.16)	1.09 (0.10)	0.00002	0.00004	0.04928	0.00172
Median (min–max)	1.02 [0.84–1.16]	1.21 [1.05–1.67]	1.11 [0.89–1.25]				
Anterior cingulate							
Mean (SD)	0.91 (0.10)	1.06 (0.10)	0.96 (0.25)	0.00093	0.00129	0.86309	0.00385
Median (min–max)	0.92 [0.65–1.07]	1.03 [0.93–1.27]	0.91 [0.64–1.81]				
Posterior cingulate							
Mean (SD)	0.96 (0.11)	1.22 (0.13)	1.06 (0.17)	0.00002	0.00005	0.04939	0.00112
Median (min–max)	0.98 [0.72–1.16]	1.19 [1.05–1.55]	1.07 [0.77–1.57]				

SUVr represents regional maximal PET intensity to cerebellum ratio.

AD: Alzheimer's disease; MCI: mild cognitive impairment.

Anterior cingulate stands for caudal anterior cingulate, rostral anterior cingulate, and isthmus cingulate cortex.
